# Family Caregiver: The Forgotten Savior

**DOI:** 10.30476/IJCBNM.2021.90118.1673

**Published:** 2021-10

**Authors:** Sima Sadat Hejazi, Meimanat Hosseini, Abbas Ebadi, Hamid Alavi Majd

**Affiliations:** 1 Student Research Committee, Department of Community Health Nursing, School of Nursing and Midwifery, Shahid Beheshti University of Medical Sciences, Tehran, Iran; 2 Department of Community Health Nursing, School of Nursing and Midwifery, Shahid Beheshti University of Medical Sciences, Tehran, Iran; 3 Behavioral Sciences Research Center, Life Style Institute, Faculty of Nursing, Baqiyatallah University of Medical Sciences, Tehran, Iran; 4 Department of Biostatistics, Department of Paramedicine, Shahid Beheshti University of Medical Sciences, Tehran, Iran

## DEAR EDITOR

The family caregivers are spouse, family members, friends, or relatives of the sick, disabled, and dependent individuals who are not paid for providing care. The care may last for months or years and covers a wide range of physical, social, emotional, or even financial care. Statistics show that more than 40 million adults in the United States care for their loved ones as family caregivers. ^1^ In Iran, no specific statistics are available on the number of caregivers of patients with chronic diseases. However, by examining the statistics of chronic diseases, as well as the trend of population aging in Iran, it is most likely that a large part of the community is involved in caring for a sick, elderly, or disabled person. 

The persistent and often physical nature of caring duties and emotional complications resulting from providing care to a loved one with a debilitating illness is considered a chronic stressor. ^2^ This stressor, which has a chronic nature, caregiver burden, and the uncertainty about the end time of the need for this care, is associated with numerous physical and psychological consequences for the caregiver. ^3^ Due to involvement in care, caregivers lose job opportunities. On the other hand, caregiving also leads to social isolation of the caregivers. ^2^ This condition is referred to as a general concept of caregiver burden. ^3^ Consequently, involvement in providing care is a public health issue that affects millions of individuals’ quality of life. ^4^

Demographic change and therapeutic advances in chronic disorders may shortly highlight the informal care. Despite the increasing need for informal care, changes in family structure and social roles in the future may endanger the potential for informal care or, at least, increase care burden. ^5^ Therefore, to take advantage of this vital and influential role of family caregivers, reduce caregiver burden, and encourage and motivate them to continue this challenging path, we must find a solution. Specific priorities for supporting family caregivers, including recognizing needs and problems, increasing awareness, supporting work environments, and healthcare, have been identified worldwide. Various studies have been conducted in different areas and based on diverse methods to assess the caregivers’ needs and burdens and evaluate the effectiveness of various interventions. In Iran, several studies have been conducted, too; however, there is no well-written program or a special center with specific goals for family caregivers that can practically utilize the results of these studies. 

Nursing is a discipline with a broad field. In all nursing areas, the emphasis is placed on caring for clients and their families. In Iran, likewise, the patient’s family has been considered in the curriculum of different nursing levels. Although nurses may focus on supporting the client’s family in their routine care programs, in the absence of an identified and responsible unit for caring for caregivers, these minor efforts will not resolve significant problems. On the other hand, nursing staff shortage issues do not allow the nurses in medical centers to care for family caregivers along with their patients. The lack of implementation of nursing care programs at the family and community level and the focus of nursing care on medical centers, and the lack of even a unit in these medical centers to assess and address the caregivers’ problems and not merely their patients complicated the situation. In the absence of such centers and the lack of micro- and macro-planning, there is no opportunity to apply the results of the studies, and none of the caregivers’ major or minor problems will be resolved.

It seems that the establishment of centers and facilities to educate, counsel, and support the caregivers of individuals with chronic conditions, such as cancer, mental disorders, Alzheimer’s, dementia, diabetes, heart failure, cerebrovascular accidents, spinal cord injuries, multiple sclerosis, and kidney failure, is of particular importance. Moreover, strengthening the relationship between the patient and family and the care-support centers through the nursing team’s home care can help support the patients’ caregivers. On the other hand, taking advantage of electronic and virtual services, creating websites, and mobile-based programs, particularly remote and less privileged areas can be beneficial. Furthermore, this group’s financial issues and the need to support them with the help of insurance services and government financial support are suggested to be considered and planned. 

## References

[1] Alam S, Hannon B, Zimmermann C ( 2020). Palliative Care for Family Caregivers. Journal of Clinical Oncology.

[2] Bastawrous M ( 2013). Caregiver burden--a critical discussion. International Journal of Nursing Studies.

[3] Carretero S, Garcés J, Ródenas F, Sanjosé V ( 2009). The informal caregiver’s burden of dependent people: Theory and empirical review. Archives of Gerontology and Geriatrics.

[4] National Association of Chronic Disease Directors (2019). Caregiving for Family and Friends - A Public Health Issue.

[5] Fekete C (2015). Caregiver Burden. Caregiver Burden. In: Wright JD. International Encyclopedia of the Social & Behavioral Sciences.

